# Clinical outcomes of self-glazed zirconia veneers produced by 3D gel deposition: a retrospective study

**DOI:** 10.1186/s12903-024-04253-2

**Published:** 2024-04-15

**Authors:** Feifei Yu, Fangyue Xiang, Jing Zhao, Nengjie Lin, Zhe Sun, Yuanna Zheng

**Affiliations:** 1 School/Hospital of Stomatology, Zhejiang Chinese Medical University, Hangzhou, Zhejiang China; 2 903 Hospital People’s Liberation Army, Hangzhou, Zhejiang China; 3 Ningbo Dental Hospital/Ningbo Oral Health Research Institute, Ningbo, Zhejiang China

**Keywords:** Veneers, Self-glazed zirconia, lithium disilicate glass–ceramics, Clinical outcomes

## Abstract

**Background:**

Self-glazed zirconia (SZ) restorations are made by a novel additive three-dimensional gel deposition approach, which are suitable for a straightforward completely digital workflow. SZ has recently been used as minimally invasive veneer, but its clinical outcomes have not been clarified yet. This study aimed to evaluate the preliminary clinical outcomes of SZ veneers compared with the widely used lithium disilicate glass–ceramic veneers made by either pressing (PG) or milling (MG) process.

**Methods:**

Fifty-six patients treated with SZ, PG, and MG veneers by 2 specialists between June 2018 and October 2022 were identified. Patients were recalled for follow-up at least 1 year after restoration. Clinical outcomes were assessed by 2 independent evaluators according to the modified United States Public Health Service (USPHS) criteria. Overall patient satisfaction was assessed using visual analogue scale (VAS), and analyzed by one-way ANOVA. Chi-square test was applied to compare the difference in the success and survival rates among the 3 groups.

**Results:**

A total of 51 patients restored with 45 SZ, 40 PG, and 41 MG veneers completed the study, with a patient dropout rate of 8.9%. Mean and standard deviation of follow-up period was 35.0 ± 14.7 months. All restorations performed well at baseline, except for 2 SZ veneers with mismatched color (rated Bravo). During follow-up, marginal discrepancy (rated Bravo) was found in 4 MG veneers and 1 PG veneer, and partially fractured (rated Charlie) was found in another 2 PG veneers. The survival rate of SZ, PG, and MG veneers was 100%, 95%, and 100%, with a success rate of 95.56%, 92.50%, and 90.24%, respectively, none of which were significantly different (*p* = 0.099 and 0.628, respectively). The mean VAS score of SZ, PG, and MG was 95.00 ± 1.57, 93.93 ± 2.40, and 94.89 ± 2.00 respectively, without significant difference (*p* > 0.05).

**Conclusion:**

SZ veneers exhibited comparable preliminary clinical outcomes to PG and MG veneers, which could be considered as a feasible option for minimally invasive restorative treatment.

## Background

Veneers, as a minimally invasive restorative technique with both aesthetic and biocompatibility, have been widely used in clinical practice [1, 2]. Compared with conventional full crowns, veneers maximize the preservation of tooth tissue, as well as offer a great aesthetic potential [3]. However, bonding and mechanical properties should be carefully considered when selecting restorative materials for veneers in order to enhance overall success. Since the limited tooth preparation for veneers does not offer enough macromechanical bonding, a strong micromechanical or adhesive bonding is crucial [4]. Meanwhile, veneers are always thin so that their strength would be compromised to some extent [5].

Glass ceramics are the prevalent materials used for producing ceramic veneers in clinic, with lithium disilicate being especially notable for its excellent translucency characteristics, good adhesion to resin cement, and higher mechanical strength than other kinds of glass ceramics [6]. Previous studies on lithium disilicate veneers have shown optimistic results, with the survival rates over 10 years were 97.4% ~ 100% [7–9]. The main complaint was fracture and debonding. In addition, Alvaro et al. [10] reported an estimated 5-year survival rate of 90.1% for 212 lithium disilicate veneers used to restore the generalized severe pathological tooth wear, with fracture being the most common complication.

Zirconia ceramics have excellent mechanical properties and biocompatibility, and they have also been used to produce veneers with improvements in aesthetics [11]. However, due to the inherent inertness, the greatest challenge lies in their low adherence to resin cement compared with glass ceramics, which can be conditioned by hydrofluoric acid and silanization [12]. Despite numerous surface treatments proposed to modify the bonding surface of zirconia, the retention of zirconia veneers remains a problem [13]. At present, there are few clinical reports on zirconia veneers, with only one case report showing that the ultrathin zirconia veneers presented very acceptable aesthetic results and satisfactory performance after one-year follow-up [14].

The fabrication of fixed dental crowns and bridges encompasses both subtractive and additive manufacturing methods, each offering unique advantages in the production process. Subtractive techniques involve the precise removal of material from solid blocks using milling machines, allowing for meticulous sculpting of dental materials such as ceramics or hybrid materials [15, 16]. Conversely, additive manufacturing, also known as 3D printing, builds up material layer by layer guided by computer-aided design (CAD) software, facilitating the creation of intricate and personalized restorations [17]. While subtractive methods excel in providing exceptional surface finish and durability, additive techniques offer rapid customization and efficiency, transforming the landscape of dental prosthetics fabrication. The integration of subtractive and additive manufacturing methodologies represents a dynamic convergence, empowering dental practitioners with a diverse array of tools to deliver precise, aesthetically pleasing, and patient-specific fixed dental prosthetics [18].

Shen et al. [19] developed a new type of self-glazed zirconia (SZ) restorations, and their manufacturing process combined both subtractive and additive manufacturing methodologies. Different from the conventional subtractive milling of the partially sintered zirconia blanks, the green body of SZ was formed by 3D additively depositing zirconia gel, followed by a milling procedure over the intaglio surface. This process imparted SZ with minimized defects, a gradient nanostructure with enamel-like outer surface, as well as good translucency, color, and profile stability [20, 21]. It has been verified that SZ had better mechanical and aesthetic properties than conventional milled zirconia [22–24]. Besides, a novel sol–gel coating technique was applied to improve the bonding properties of SZ during manufacturing. In vitro studies showed that the bonding strength of sol–gel coated SZ was comparable to the etched and silanized lithium disilicate glass–ceramics, and was significantly higher than sandblasted conventional milled zirconia [25, 26].

Ren et al. [27] first applied SZ veneers to repair the chipped porcelain-fused-to-metal restorations and obtained ideal aesthetic and functional effects after 12 months. Ding et al. [28] successfully applied SZ ultra-thin veneers to restore the excessive diastema without grinding teeth, while further follow-up was lacking. So far, there are few studies available on the clinical evaluation of the novel SZ veneers. This retrospective study aimed to assess the preliminary clinical outcomes of SZ veneers in comparison with the commonly used pressed- and milled glass–ceramic (PG and MG) veneers. The null hypothesis was that there is no difference in survival or success rate among the different types of veneers.

## Methods

### Study population

Patients who received SZ, PG, and MG veneers in the Stomatological Hospital affiliated to the Zhejiang Chinese Medical University between June 2018 and October 2022 were included. This study complied with the Helsinki Declaration and was approved by the Institutional Review Board of the Stomatological Hospital Affiliated to the Zhejiang Chinese Medical University (#202,205,210,005). All patients signed the informed consent for the clinical study. The inclusion criteria were as follows: patients were over 18 years old with good oral hygiene and low caries activity; vital tooth with more than 50% preserved enamel needed to be restored; and teeth restored with veneers due to the esthetic deficits, including correction of contour and size, diastemas, tooth wear and other noncarious dental tissue loss, tooth misalignment, and/or limited tooth discoloration. Patients with uncontrollable parafunctional movement or periodontal disease, as well as those with obvious mental illness or serious systemic disease were excluded.

### Prosthetic procedures

All the clinical procedures were performed by two independent prosthodontists (Y.Z. and L.W.), each with more than 10 years of experience, and a dental technician with at least 3 years of experience. Patients in all three groups were scheduled for two visits, approximately one week apart. Firstly, a shade guide (VITA 3D-Master, VITA Zahnfabrik, Bad Sackingen, Germany) was used to select tooth shade, and digital photographs were taken to record the aesthetic characteristics. When tooth preparation was necessary, the medium and fine grit diamond rotary instruments (DIA-BURS, MANI, Utsunomiya City, Japan) with high-speed handpiece were used. According to the manufacturers, the minimum thickness of SZ on the axial surfaces and on the occlusal surface should be no less than 0.3 mm and 0.8 mm, respectively, and the minimum thickness of PG and MG on the axial surfaces and on the occlusal surface should be no less than 0.5 mm and 1.0 mm, respectively. For labial veneers, the gingival margin was designed as a feather or light chamfer, and was placed at the gingival lever or 0.5 mm subgingivally. While for occlusal veneers, the margin was carefully planned to prevent any contact with the opposing teeth during occlusion [29, 30]. The feather-edged margins were applied for optimal preservation of healthy tooth structure, enhancing fracture resistance and tooth bonding [4, 31], with proven good clinical performance [32–34]. The abutment teeth were prepared within the enamel, and the internal line angle of the preparation was smooth and round. A single-cord or double-cord gingival displacement was applied for gingival retraction. Digital casts were obtained by intraoral scanning (iTero Element 2, Zimmer Dental, Florida, USA), and the quality of the preparation was assessed. The interim restoration was designed by computer software (exocad, exocad GmbH, Darmstadt, Germany), and produced by a chairside milling machine (Ardenta CS100-5W, ARIX CNC Machines CO., Ltd., Germany) with denture resin (PMMA Disk, YAMAHACHI DENTAL MFG., CO., China). The seating, morphology, occlusion, marginal adaption, and proximal contacts of the interim veneers were clinically checked and adjusted if needed. In this study, the interim veneers were mainly used to evaluate the aesthetics and fit of the proposed final restorations. Most of interim veneers did not require bonding. However, when bonding was needed, two cements could be chosen according to different preparation designs of veneers. Zinc polycarboxylate cement is pulp-friendly and easy to clean [35], which could be used for temporary bonding of veneers with incisal overlap preparation. For the veneers prepared by window, feather or bevel, they were cemented with a point adhesion technique due to the poor retention [36].

For fabricating SZ veneers, the design files of the interim restorations were adjusted according to the results of clinical try-in, and then sent to the manufacturer [37]. SZ veneers (Self-glazed zirconia, Erran Tech, Hangzhou, China) were produced by additive wet 3D gel deposition technique. Firstly, the green bodies of the restorations were formed by wet deposition of 3 mol% Y_2_O_3_ partially stabilized ZrO_2_ (3Y-TZP) gel, and then the intaglio surfaces and margins were green milled. Subsequently, a hybrid gel, comprising aluminum nitride and aluminum hydroxide powders, was prepared for sol–gel coating, resulting in a film of approximately 0.5 to 2 µm thickness on the intaglio surface [26]. After drying, the restorations were sintered at 1450 °C for 90 min. The veneers were cleaned and ready for use in the clinic without glazing.

**Fig. 1:**
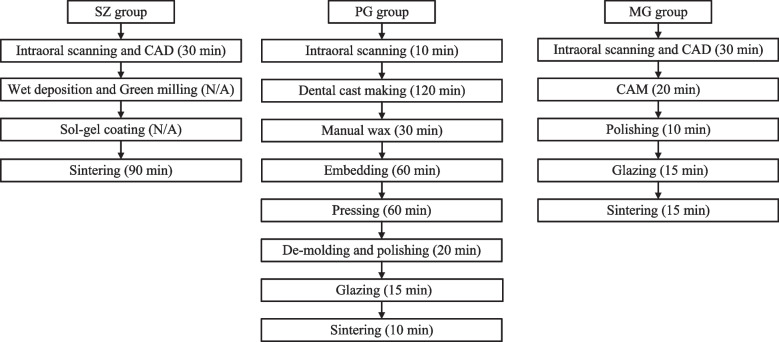
The recommended production process and time for fabricating a single unit of SZ, PG, and MG veneers by the manufacturers. N/A: The processing time not disclosed since these procedures are regarded as central production elements and trade secrets. *CAD* Computer Aided Design, *CAM* Computer Aided Manufacturing

For fabricating PG and MG veneers, digital casts were sent to the manufacturer. The PG veneers were produced by the lost-wax technique. Firstly, the digital casts were printed out and the wax patterns were made manually, then the restorations were made with glass–ceramic ingots (IPS e.max Press lithium disilicate, Ivoclar Vivadent, Schaan, Liechtenstein) in a pressing system (Programat EP 5000, Ivoclar Vivadent, Schaan, Liechtenstein) at 915 °C for 60 min. After glazing (IPS e.max Ceram, Ivoclar Vivadent, Schaan, Liechtenstein), the restorations were sintered in a furnace (P300, Ivoclar Vivadent, Schaan, Liechtenstein) at 700 °C for 10 min. MG veneers were designed digitally and then fabricated by subtractive dry-milled of DiSiLi blocks (IPS e.max CAD, Ivoclar Vivadent, Schaan, Liechtenstein) using a dental CAD/CAM system (CEREC 3D system, Sirona, Bensheim, Germany). After glazing (IPS e.max CAD Crystall/Glaze Paste, Ivoclar Vivadent, Schaan, Liechtenstein), the restorations were sintered in a furnace at 840 °C for 15 min. Figure 1 shows the recommended production process and time for fabricating a single unit of SZ, PG, and MG veneers by the manufacturers.

**Fig. 2:**
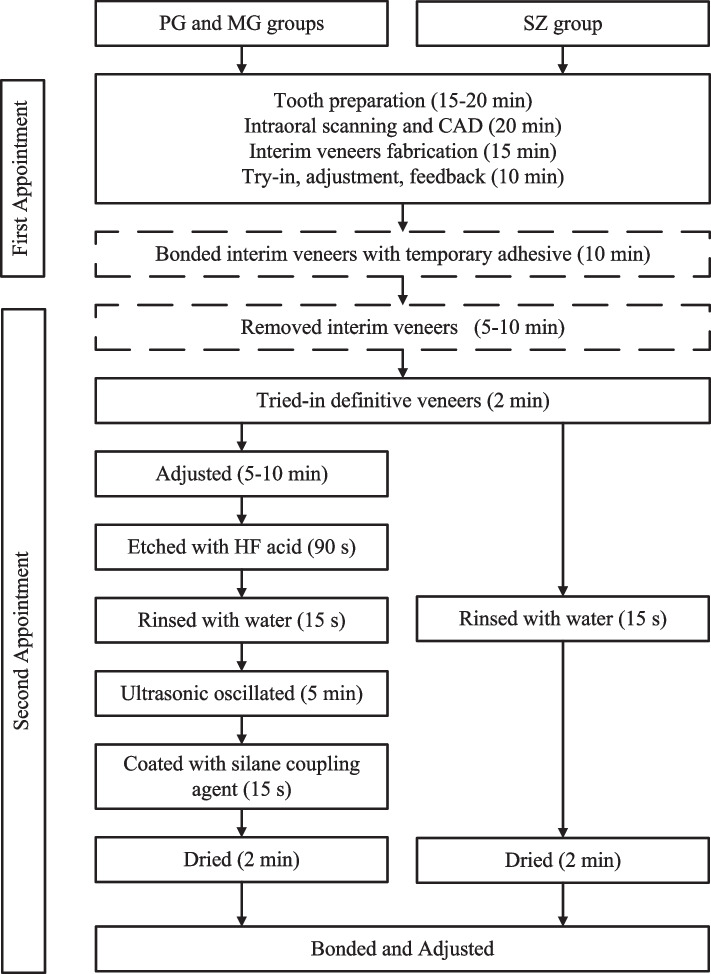
The recommended clinical restoration process and time for a single unit of SZ, PG, and MG veneers by the manufacturers. Dashed box: non-essential operations

**Fig. 3:**
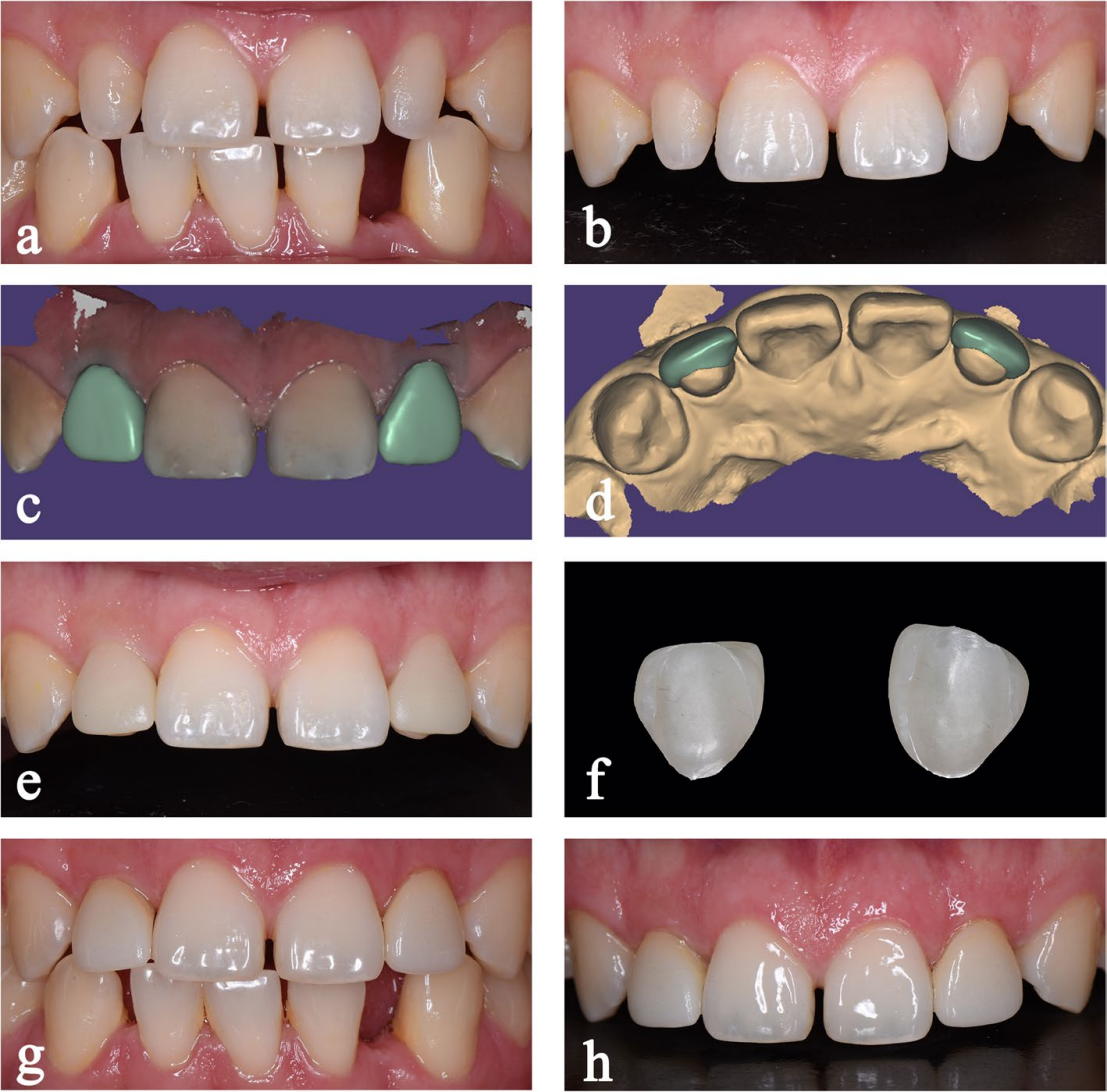
Minimally invasive restorative process of the teeth 12,22 with self-glazed zirconia veneers. Preoperative clinical photos: **a**, intraoral frontal view of the maxillary anterior teeth, and **b**, frontal view. **c** and **d**, digital design of the veneers. **e**, frontal view of the maxillary anterior teeth with cemented interim veneers. **f**, self-glazed veneers produced by the additive 3D gel deposition technique. Postoperative clinical photos: **g**, intraoral frontal view of the maxillary anterior teeth, and **h**, frontal view

During the 2nd visit, typically scheduled one week after the 1st visit, the interim restorations were first removed, and then the preparation surfaces were cleaned. The definitive veneers were then tried-in and adjusted as necessary before being cemented according to different protocols. The rubber dam isolation was employed in most cases (93.7%). For patients suffering from rhinitis who cannot cooperate with nasal breathing, as well as those who strongly reject rubber dams due to psychological factors, alternative isolation methods such as retractors, a high-speed suction system, and Teflon tapes were utilized. The bonding surface of PG and MG veneers was etched with 9.5% HF acid (Porcelain Etch, Ultradent, Utah, USA) for 90 s, and rinsed with water for 15 s, then ultrasonic oscillated for 5 min, followed by coated with silane coupling agent (Monobond N, Ivoclar Vivadent, Schaan, Liechtenstein) and dried for 2 min. While the bonding surface of the SZ was just rinsed with water for 15 s and dried for 2 min. Figure 2 shows the recommended clinical restoration process and time for a single unit of SZ, PG, and MG veneers by the manufacturers. The tooth surface was etched with 35% phosphoric acid (Scotchbond Universal Etchant, 3 M ESPE, Minnesota, USA) for 15 s, thoroughly rinsed with water for 10 s, and then dried. Adhesive (Scotchbond Universal Adhesive, 3 M ESPE, Minnesota, USA) was applied to both the restorations and tooth surfaces for 20 s and dried with air. A resin cement (RelyX Ultimate, 3 M ESPE, Minnesota, USA) was applied for bonding. After a 1200 MW light-curing (Elipar Free-light, 3 M ESPE, Minnesota, USA) for 3 s, the excess cement was wiped off, and a further 20 s of light curing was conducted. The occlusion was refined as needed, the static and dynamic occlusion (including protrusion and laterality) was assessed using 100 μ m blue occlusal paper and 40 μ m red occlusal paper (Dental Articulating Paper, Shanghai Rongxiang Dental Co., China). This allowed for the detection and elimination of potential occlusal disturbances until achieving uniform contact. Following adjustments, the veneers were polished using a general-purpose ceramic polishing kit (Diacera RA, EVE, Pforzheim, Germany). Figure 3 illustrates a typical minimally invasive veneer restorative process, which maximizes the preservation of tooth structure during the preparation and finishing procedure.

### Clinical evaluation

Patients were recalled at least 1 year after restoration. The quality of restorations regarding color match, anatomic form, integrity, retention, secondary caries, marginal adaptation, and discoloration, was evaluated by two independent evaluators (F.Y. and N.L.) following the modified United States Public Health Services (USPHS) criteria [38] at baseline and at follow-up time point. The restorations were rated as Alfa (A), Bravo (B), Charlie (C). Survival was defined as the absence of clinically unacceptable ceramic fracture or a biological event (such as caries, poor marginal adaptation, or severe marginal discoloration) that required replacement of the entire restoration or extraction of the tooth. Success was defined as a restoration remaining unchanged throughout the observation period and not requiring any intervention to maintain function. In cases of disagreement, a consensus was reached by discussion after reviewing the rating criteria. The Cohen’s Kappa assessment was carried out to determine interrater agreement.

In addition, patients were asked to rate their overall satisfaction with the restorations using a visual analogue scale (VAS) on a 100 mm long horizontal scale (the higher the score indicated greater satisfaction), and whether they had any sensitivity or discomfort after restoration.

### Statistical analysis

Descriptive statistics were performed for qualitative variables. Data analysis was performed using SPSS (IBM SPSS Statistics 25.0, Armonk, NY, USA), and means and standard deviations were calculated for the variable of patient satisfaction. Chi-square tests were applied to test for differences between the 3 groups in success and survival rates, and likelihood ratio (LR) tests were used if the assumptions of the chi-square test were invalid. One-way ANOVA was used to compare the values of VAS (α = 0.05).

## Results

A total of 56 patients who received veneers were enrolled, 5 of whom (8.9%) were unable to attend appointments due to personal reasons. Therefore, 51 patients with 126 veneers were included in the present study, including 45 SZ, 40 PG, and 41 MG. The mean follow-up time was 35 months with a standard deviation of 14.7 months. The mean age of patients was 35 years, ranging from 18 to 66 years with a female predominance. Poor aesthetics (26%), dental trauma (24%) and tooth defects (24%) were the main complaints for restoration. The distribution of restorations is listed in Table 1, which shows that the percentages of labial veneers and occlusal veneers were 84.13% and 15.87%, respectively. Moreover, maxillary incisor veneers accounted for the largest proportion of all restorations and most were in female patients.

**Table 1:** Distribution of the 3 kinds of veneers by locations and types (*N* = 126)

Group	Location	Incisor		Canine		Premolar		Molar	
		Male	Female	Male	Female	Male	Female	Male	Female
SZ (*n* = 45)	Maxillary	13	16	0	4	0	2	0	0
	Mandibular	0	0	0	2	0	3	0	5
PG (*n* = 40)	Maxillary	9	15	0	4	0	2	0	0
	Mandibular	0	5	0	3	0	1	0	1
MG (*n* = 41)	Maxillary	11	16	0	2	0	3	0	0
	Mandibular	0	4	0	2	0	3	0	0

The Cohen’s Kappa between the 2 evaluators was 0.757, indicating reliable agreement. During try-in, 2 SZ group (4.4%) needed minimal occlusal adjustments, including 1 anterior veneer and 1 posterior occlusal veneer. In contrast, 10 MG anterior veneers and 15 PG anterior veneers had occlusal adjustment, with adjustment rates of 25.0% and 36.6%, respectively. Table 2 presents the preliminary clinical outcomes of SZ, PG, and MG groups according to USPHS criteria. All restorations showed excellent performance at baseline, except for 2 SZ veneers, which were rated Bravo due to their excessive translucency and yellowish color after cementation with yellow adhesive. At the follow-up time point, 2 SZ veneers were still yellowish. Four MG veneers and 1 PG were rated Bravo due to marginal discrepancies, with the latter exhibiting bleeding on probing. In addition, 2 PG veneers were rated Charlie due to partial fracture, one of which had deep secondary caries and proximal tooth structure defect. No debonding or marginal discoloration occurred in restorations at the follow-up examination (Table 3). The survival and success rates are listed in Table 4. No significant differences in success and survival rates among the 3 types of veneers were found (*p* > 0.05).

**Table 2:** Clinical outcomes of 3 kinds of veneers evaluated at baseline and at follow-up time point (mean, 35 months)

Evaluation criteria	SZ (n, %)						PG (n, %)						MG (n, %)					
	Baseline			Follow-up			Baseline			Follow-up			Baseline			Follow-up		
	A	B	C	A	B	C	A	B	C	A	B	C	A	B	C	A	B	C
Color match	43 (95.56)	2 (4.44)	0	43 (95.56)	2 (4.44)	0	40 (100)	0	0	40 (100)	0	0	41 (100)	0	0	41 (100)	0	0
Anatomic form	45 (100)	0	0	45 (100)	0	0	40 (100)	0	0	40 (100)	0	0	41 (100)	0	0	41 (100)	0	0
Restoration integrity	45 (100)	0	0	45 (100)	0	0	40 (100)	0	0	38 (95)	0	2 (5)	41 (100)	0	0	41 (100)	0	0
Restoration retention	45 (100)	0	0	45 (100)	0	0	40 (100)	0	0	40 (100)	0	0	41 (100)	0	0	41 (100)	0	0
Marginal adaptation	45 (100)	0	0	45 (100)	0	0	40 (100)	0	0	39 (97.50)	1 (2.50)	0	41 (100)	0	0	37 (90.24)	4 (9.76)	0
Marginal discoloration	-	-	-	45 (100)	0	0	-	-	-	39 (100)	0	0	-	-	-	41 (100)	0	0
Secondary caries	-	-	-	45 (100)	0	0	-	-	-	39 (97.50)	0	1 (2.50)	-	-	-	41 (100)	0	0

**Table 3:** Details and figures of unsuccessful veneers

Group	Tooth Position	Follow-up Time	Complication	Image
SZ	11,21	37 months	yellowish color after cementation with yellow resin cement	
MG	11,21,31,41	27 months	incompatible margins and bleeding on probing	
PG	21	24 months	Slight probe catching without gap (black arrow)	
PG	46	30 months	Veneer fractured, secondary caries and the proximal tooth structure defect were found

**Table 4:** Survival and success rates of the 3 kinds of veneers and the patients’ overall satisfaction

Groups	Survival			Success			Overall satisfaction		
	n (%)	x^2^	*P* value	n (%)	x^2^	*P* value	VAS (mean ± SD)	F	*P* value
SZ	45 (100)	2.894	0.099	43 (95. 56)	0.999	0.628	95.00 ± 1.57	2.228	0.112
PG	38 (95)			37 (92.50)			93.93 ± 2.40		
MG	41(100)			37(90.24)			94.89 ± 2.00		

Patients’ overall satisfaction with the 3 types of veneers is presented in Table 4. There was no statistically significant difference between PG and MG (*p* = 0.112), although the VAS value of SZ group was relatively higher than the other 2 groups.

## Discussion

The null hypothesis of the present study was accepted, as the success and survival rates of SZ, PG, and MG veneers in a follow-up period of 35.0 ± 14.7 months, as well as their overall patient satisfaction, were found to be similar and high.

The study did not observe any significant issues with the debonding of zirconia veneers, which was the primary concern. It indicated that the bonding surface modification was effective. Sol–gel coating technique endowed an irregular porous structure on the restorations’ bonding surface, which was conducive to improving the micromechanical bonding strength and reducing the risk of veneers loss [25, 26]. Meanwhile, no fracture or chipping of SZ veneers was observed in the present study, which was beneficial to the unique processing technology and excellent mechanical properties [39]. Furthermore, SZ veneers exhibited good marginal adaptation, with no evidence of marginal discoloration or secondary caries during follow-up. Compared with conventional milled zirconia, SZ could achieve superior dimensional accuracy and margin quality [17, 40].

Two SZ veneers were deemed unsuccessful due to color mismatch in the present study. In this case, ultra-thin veneers were adopted for the two maxillary central incisors to close maxillary midline diastema, and teeth preparation was avoided for the sake of protecting of abutment teeth. Considering the normal translucency and color of the abutment teeth, we opted for high-translucent SZ material as recommended by the manufacturer. The color and translucency of veneers were matched to adjacent teeth during tried-in, however, they looked yellowish after cementation. It could be ascribed to the color exposure of cement [41]. The color change effect of resin cement increases when the ceramic thickness decreases, especially for the high-translucent veneers [42]. The try-in paste was not used during restoration because the color of try-in paste was always inconsistent with the resin cement [43, 44]. Moreover, the materials and thickness of the restorations could affect the agreement of try-in paste and the respective resin cement [45]. Therefore, an adhesive system for high-transparency ultra-thin veneers, including color-matched try-in paste and resin cement, is intentional. Alternatively, choosing SZ veneer with a slightly lower transparency, or a combination of high translucency and internal color masking technology may also be effective solutions.

The success and survival rates of PG and MG veneers in this study were in agreement with the previous studies [32, 46]. Different processing techniques had no effect on the performance of lithium disilicate veneers [47]. All lithium disilicate veneers showed satisfactory performance at baseline, but some problems were found at the follow-up time point. One PG occlusal veneers used to restore the defects of the mandibular right first molar was partial fracture with significant secondary caries and dental tissue defect. Additionally, at the 61-month follow-up, it was found that another PG anterior veneer had a fractured proximal mesial angle and could not be repaired directly. Considering the high biting force in the posterior region and the limited space for minimally invasive restoration, it is recommended to select ceramic materials with higher strength than lithium disilicate [46, 48]. In addition, a PG veneer used to restore a defective maxillary left mesial incisor revealed a slight marginal discrepancy after 24 months. This is similar to another unsuccessful case where four maxillary and mandibular anterior MG veneers were also found marginal discrepancies after 27 months, along with gingival bleeding on probing. This could be correlated with the cement wear and marginal deterioration of the luting space [49]. Better marginal fit could minimize the risk of cement wear [47].

Patients’ satisfaction was considered as one of the most important criteria for assessing clinical success. The results of the present study demonstrated high levels of patient satisfaction with the 3 kinds of veneers, which were very similar to the previous studies [10, 48]. Some patients in the MG and PG groups complained that the clinical try-in and bonding procedures were time-consuming. This may be due to the fact that many MG and PG veneers need to undergo grinding and polishing, which typically took about 5–10 min. However, such complaints were rare for SZ veneers. On the one hand, SZ veneers were manufactured with a completely digital process, ensuring accuracy through intraoral try-in and adjustment of the temporary restorations, while eliminating manual operations to reduce errors. Therefore, the need for adjustment of SZ definitive restorations was minimized [37]. On the other hand, the pretreatment of the bonding surface of SZ was done during the manufacturing process, and no further treatment was required clinically [28]. But the cost of SZ veneers raised concerns among some patients as its price was approximately 50% higher than that of MG and PG veneers.

Limitations of this retrospective study included a small sample size and a relatively short follow-up period. Mean follow-up period was just 35 months, which could only provide preliminary findings. A well-designed large-scale clinical study is needed to clarify the long-term performance of SZ veneers.

## Conclusions

Within the limitations of this retrospective study, it can be concluded that SZ, PG, and MG veneers had high success rates, survival rates, and overall patient satisfaction in the short-term follow-up observations. The novel SZ veneers showed comparable preliminary clinical outcomes to the widely used PG and MG veneers, which offered a reliable alternative for minimally invasive clinical restoration.

## Data Availability

Data are available upon reasonable request. Data can be available by contacting the corresponding author.

## Abbreviations

USPHS: United States Public Health Service
VAS: Visual Analogue Scale
SD: Standard Deviation
